# Athletes’ basic psychological needs and emotions: the role of cognitive reappraisal

**DOI:** 10.3389/fpsyg.2023.1205102

**Published:** 2023-07-13

**Authors:** Claudio Robazza, Milena Morano, Laura Bortoli, Montse C. Ruiz

**Affiliations:** ^1^BIND-Behavioral Imaging and Neural Dynamics Center, Department of Medicine and Aging Sciences, “G. d’Annunzio” University of Chieti-Pescara, Chieti, Italy; ^2^Parisi-De Sanctis Institute, MIUR (Italian Ministry of Education, University and Research), Foggia, Italy; ^3^School of Medicine and Health Sciences, “G. d’Annunzio” University of Chieti-Pescara, Chieti, Italy; ^4^Faculty of Sport and Health Sciences, University of Jyväskylä, Jyväskylä, Finland

**Keywords:** self-determination theory, process model of emotion regulation, cognitive reappraisal, expressive suppression, psychobiosocial experiences

## Abstract

In sport, where high achievements are at stake, athletes often feel pressure and emotions that hinder their performance. Emotion regulation becomes essential for athletes to handle stress, achieve optimal performance, and enhance their overall well-being. To advance both research and practical applications, it is crucial to examine the antecedents of emotion regulation and the impact on emotions and other feelings associated with performance. Specifically, the purpose of this cross-sectional study was to examine the role of athletes’ emotion regulation strategies (i.e., cognitive reappraisal and expressive suppression) in the relationship between basic psychological needs satisfaction, emotions, and psychobiosocial experiences. The sample consisted of 424 competitive athletes (246 men and 178 women) involved in individual sports (*n* = 164; e.g., fencing, gymnastics, martial arts, swimming, and tennis) or team sports (*n* = 260; e.g., basketball, rugby, soccer, and volleyball), aged 16–36  years (*M* = 23.08, *SD* = 7.65). Their competitive experience ranged from 1 to 21  years (*M* = 9.71, *SD* = 6.34) at regional (71%), national (18%), or international (11%) level, and they practiced their sport on average 3.74 times a week (*SD* = 1.73). Participants completed measures of basic needs satisfaction (i.e., competence, autonomy, and relatedness), emotion regulation style, emotions, and psychobiosocial experiences. Structural equation modeling results showed that competence need satisfaction was positively associated with pleasant emotions and psychobiosocial experiences that are perceived as functional for performance, and negatively associated with a maladaptive emotion regulation style (i.e., expressive suppression) and unpleasant emotions. Relatedness need satisfaction was positively related to an adaptive emotion regulation style (i.e., cognitive reappraisal), pleasant emotions, and psychobiosocial experiences, and negatively related to expressive suppression and unpleasant emotions. Finally, mediation analysis showed positive indirect effects from autonomy and relatedness satisfaction to pleasant emotions and psychobiosocial experiences via cognitive reappraisal. Findings suggest that the satisfaction of athletes’ basic psychological needs of autonomy and relatedness is related to the experience of pleasant emotions and functional psychobiosocial states when they adopt an adaptive emotion regulation style.

## 1. Introduction

Perceived pressure and dysfunctional emotions are often experienced in many professional, artistic, and sporting contexts where high achievements are at stake (Guyon et al., 2020; Milne and Neely, 2022; Furley et al., 2023). Emotion regulation is crucial to successfully dealing with stressful situations, attaining good performance, and maintaining or improving physical and psychological health (Ruiz and Robazza, 2020). For research and applied purposes, it is therefore important to investigate the antecedents of emotion regulation (such as basic psychological needs satisfaction) and the consequences on emotions and other feelings related to performance.

Gross’s (1998, 2014, 2015) process model of emotion regulation has attracted a wide research interest so far and stimulated investigation into individual strategies used to manage the occurrence, intensity, duration, and experience of emotions (Hu et al., 2014). Five families of emotion regulation strategies are hypothesized to intervene at different points in the emotion-generative process, namely, situation selection, situation modification, attention deployment, cognitive change, and response modulation. These emotional regulation processes can involve conscious effort and can also occur without awareness (Gyurak et al., 2011).

Cognitive reappraisal is one of the most investigated antecedent-focused cognitive change strategies of the process model that occurs before the emotional response has been fully activated (Uphill et al., 2012; Balk et al., 2013). An athlete, for example, can reappraise a forthcoming competitive event as a challenge rather than a threat, and perceive the competition as an opportunity to broaden their range of experiences, thereby changing the emotional impact of a previously perceived potentially harmful situation (Lazarus, 2000; Robazza et al., 2008; Sammy et al., 2021). Reappraisal has usually been reported to be an adaptive style associated with high levels of pleasant affect (e.g., Balzarotti et al., 2010; Uphill et al., 2012; Ioannidis and Siegling, 2015), enhanced interpersonal functioning (e.g., Cabello et al., 2013), and well-being (Levin and Rawana, 2022).

Unlike cognitive reappraisal, which is an antecedent-focused strategy, expressive suppression is a response-focused strategy in which an individual exerts effortful control and inhibits emotion response tendencies (Gross and John, 2003). For instance, before a major competition, an athlete may try to hide their apprehension about a possible poor performance to avoid being judged as weak by their coach and teammates. High expressive suppression has generally been considered a maladaptive style linked to dysfunctional emotions (Cece et al., 2019; see Preece et al., 2020).

Researchers specifically examining cognitive reappraisal and expressive suppression use in sport have found that cognitive reappraisal was positively associated with mental well-being (Bird et al., 2023) and greater experiences of pleasant emotions (Uphill et al., 2012). Similar results were reported for young athletes, showing that more favorable levels of outcome variables (i.e., higher pleasant emotions, enjoyment, confidence, satisfaction, social connection, and lower unpleasant emotions and emotional loneliness) were associated with greater use of cognitive reappraisal and less use of expressive suppression (Kim and Tamminen, 2023).

The results of two studies are particularly relevant to the present investigation. In a sample of college students, Benita et al. (2020) examined the effects of integrative emotion regulation (an adaptive emotion regulation style conceptually similar to cognitive reappraisal) and suppressive emotion regulation (a maladaptive style) on well-being. Along with the process model of emotion regulation (Gross, 1998, 2015), Benita et al.’s study relied on the basic psychological needs theory as conceptualized within the broader framework of the self-determination theory (Ryan and Deci, 2017, 2020). The basic psychological needs theory underscores the importance of satisfying the three basic psychological needs for competence, autonomy, and relatedness to enhance individual motivation and well-being (Ryan and Deci, 2017; Vansteenkiste et al., 2023). According to Ryan and Vansteenkiste (2023), these three needs are central to self-determination theory that was “…initially focused on intrinsic motivational processes, with intrinsic motivation defined as activity that is motivated (energized and directed) by its inherent satisfactions.” (p. 9). Intrinsic motivation for any activity requires a sense of autonomy (feeling in control of one’s own life), competence (feeling capable of completing a task), and relatedness (feeling part of a caring environment). When these needs are met, people are more likely to be intrinsically motivated.

Competence is conceived as a perception of mastery, a belief that one can progress and succeed. It is fulfilled in contexts that offer optimal challenges, positive feedback, and opportunities for growth. Competence frustration can lead to feelings of failure and helplessness, especially when an individual is struggling to learn or master a task. Autonomy refers to a feeling of initiative and control over one’s actions. It is enhanced by the perception of interest and value in one’s initiatives, while it is undermined by experiences of external control. Autonomy frustration involves a sense of pressure and inner conflict, a feeling of being pushed in an undesired direction, and a lack of consideration for one’s own preferences and choices. Relatedness refers to a sense of belonging and connection, which is enhanced by the expression of respect and consideration. When this need is frustrated, it leads to feelings of social isolation, exclusion, and loneliness (Vansteenkiste et al., 2020; Vansteenkiste and Soenens, 2023). Empirical evidence supports the conceptual distinction between need satisfaction and need frustration, indicating that both sets of experiences are distinct and negatively correlated (see Vansteenkiste et al., 2023). Benita et al. (2020) showed that the satisfaction of the three basic psychological needs was positively related to integrative emotion regulation and well-being, while the frustration of the same basic psychological needs was positively linked to suppressive emotion regulation and negatively associated with well-being. The results align with a growing body of research in sport that confirms (a) a positive relationship between basic needs satisfaction and adaptive sport outcomes, such as intrinsic motivation, enjoyment, well-being, physical health, behavioral engagement, and improved performance, and (b) a negative relationship between need satisfaction and maladaptive outcomes, such as burnout, exhaustion, disaffection, and unpleasant emotions (for reviews, see Schüler et al., 2023; Standage, 2023).

In a study with a sample of athletes, Robazza et al. (2022) investigated the relationships between athletes’ perceived motivational climate created by the coach, emotion regulation strategies, pleasant and unpleasant emotions (i.e., excitement, happiness, anxiety, dejection, and anger; Jones et al., 2005), and discrete emotion-related (i.e., psychobiosocial) experiences that are perceived as functional for performance (Robazza et al., 2021). Theoretical frameworks were the achievement goal theory (Nicholls, 1984), which shares assumptions and notions with basic psychological needs theory (Ryan and Deci, 2017), process model of emotion regulation (Gross, 1998), and individual zones of optimal functioning (IZOF) model (Hanin, 2007). Drawing from the IZOF model, individual psychobiosocial experiences (or states) are described as consisting of psychological (e.g., unpleasant/pleasant emotion, confidence, motivation), biological (e.g., bodily responses), and social dimensions (e.g., social support; see Robazza et al., 2021; Ruiz et al., 2021b). Psychobiosocial experiences reflect the range of emotional and non-emotional manifestations of athletes’ functioning in practice and competition (for reviews, see Ruiz and Robazza, 2020). Robazza et al. (2022) found that athletes’ perceived mastery climate, in which the coach values individual efforts, task commitment, and improvements, was positively linked to cognitive reappraisal, pleasant emotions (i.e., excitement and happiness), and psychobiosocial experiences that were perceived as functional for performance. In contrast, athletes’ perceived performance climate, where the emphasis is placed on winning and outperforming others, was positively associated with expressive suppression and unpleasant emotions (i.e., dejection and anger). Importantly, structural equation modeling showed positive indirect effects via reappraisal in the relation between perceived mastery climate and pleasant emotions/functional experiences. Positive indirect effects through expressive suppression were also observed in the relation between performance climate and unpleasant emotions.

### 1.1. Study purpose

Studies focusing specifically on cognitive reappraisal and expressive suppression in sport are scarce, even though Gross’s (1998, 2015) process model represents a prominent approach to emotion regulation. Furthermore, the role of emotion regulation in the relationship between satisfaction of basic psychological needs and emotions with their associated manifestations (i.e., psychobiosocial experiences), has not yet been studied. The present study aimed to fill this gap in the literature by focusing on athletes’ perception of psychological needs satisfaction, emotion regulation, and emotional outcomes. Specifically, based on the results of Benita et al. (2020) and Robazza et al. (2022), the main aim of this investigation was to examine the role of athletes’ emotion regulation strategies (i.e., cognitive reappraisal and expressive suppression) in the relationship between basic psychological needs satisfaction, selected emotions (i.e., happiness, excitement, anxiety, dejection, and anger), and a broad range of psychobiosocial experiences that are perceived as functional for performance. Examining relevant antecedents (i.e., basic psychological needs satisfaction) and adaptive outcomes of emotion regulation (i.e., emotions and psychobiosocial experiences) can contribute to the extant knowledge on the process model and provide practical indications to improve athletes’ well-being and performance.

Drawing from the tenets of the basic psychological needs theory (Ryan and Deci, 2017), the process model of emotion regulation (Gross, 1998), and the IZOF model conceptualization of psychobiosocial states (Hanin, 2007), we predicted that basic psychological needs satisfaction would be positively related to cognitive reappraisal, pleasant emotions (i.e., happiness, excitement), and functional psychobiosocial experiences, and negatively linked to expressive suppression and unpleasant emotions (anxiety, dejection, and anger; Hypothesis 1). Cognitive reappraisal was predicted to be positively associated with pleasant emotions/functional experiences and negatively linked to unpleasant emotions. Expressive suppression was expected to be negatively related to pleasant emotions/functional experiences and positively linked to unpleasant emotions (Hypothesis 2). Most importantly for the present study, we expected to observe indirect effects in the relationship between basic psychological needs and pleasant emotions, as well as between basic needs and functional experiences, via cognitive reappraisal (Hypothesis 3).

## 2. Method

### 2.1. Participants

The initial sample consisted of 430 competitive athletes from the main sport clubs in central Italy. After outlier removal, the final sample (*N* = 424) encompassed 246 men (89 from individual sports and 157 from team sports) and 178 women (75 from individual sports and 103 from team sports), aged 16 to 36 years (*M* = 23.08, *SD* = 7.65). The athletes had between 1 to 21 years of competitive experience (*M* = 9.71, *SD* = 6.34) at regional level (71%), national level (18%), or international level (11%). They were involved in individual sports (*n* = 164; e.g., fencing, gymnastics, martial arts, swimming, and tennis) or team sports (*n* = 260; e.g., basketball, rugby, soccer, and volleyball; see Supplementary Table 1). The participants practiced their sport an average of 3.74 times a week (*SD* = 1.73).

### 2.2. Measures

#### 2.2.1. The Basic Needs Satisfaction in Sport Scale

The Basic Needs Satisfaction in Sport Scale (BNSSS; Ng et al., 2011) is intended to assess Competence, Autonomy-choice, Internal perceived locus of causality, Volition, and Relatedness. The focus of the current study was on the three basic psychological needs of competence, autonomy, and relatedness. Therefore, we used the subscales of Competence (5 items; e.g., “I am skilled at my sport”), Autonomy-choice (4 items; e.g., “In my sport, I get opportunities to make decisions”), and Relatedness (5 items; e.g., “In my sport, there are people who I can trust”). Responses are rated on a 7-point Likert scale ranging from 1 (*not true at all*) to 7 (*very true*). Previous research by Morano et al. (2020a) supported the factor structure of the Italian version of the scale administered to a sample of athletes, showing acceptable internal consistency (ω coefficients) for Competence (0.835), Autonomy-choice (0.831), and Relatedness (0.805).

#### 2.2.2. The Emotion Regulation Questionnaire

The Emotion Regulation Questionnaire (ERQ; Gross and John, 2003) was developed to assess the use of cognitive reappraisal (6 items; e.g., “I control my emotions by changing the way I think about the situation I’m in”) and expressive suppression (4 items, e.g., “When I am feeling negative emotions, I make sure not to express them”) in samples of undergraduate students. The original stem of the items was modified from “how you control (that is, regulate and manage) your emotions” to “how you control (that is, regulate and manage) your emotions in your sporting context.” Ratings were provided on a 4-point scale ranging from 1 (*not at all*) to 4 (*very much*). Previous research has supported the factor structure of the Italian version of the scale administered to athletes, showing good internal consistency with ω values of 0.785 for reappraisal and 0.648 for suppression (Robazza et al., 2022).

#### 2.2.3. The Sport Emotion Questionnaire

The Sport Emotion Questionnaire (SEQ; Jones et al., 2005) measures the intensity of athletes’ precompetitive anxiety (5 items; e.g., “apprehensive”), dejection (5 items; e.g., “unhappy”), anger (4 items; e.g., “annoyed”), excitement (4 items; e.g., “enthusiastic”), and happiness (4 items; e.g., “joyful”). Ratings on a 5-point scale range from 0 (*not at all*) to 4 (*extremely*). The factor structure and reliability (*α* range = 0.741–0.863, composite reliability range = 0.742–0.864) were supported for the Italian version of the scale administered to athletes (Robazza et al., 2016). In the present study, we modified the question “how you feel right now, at this moment, in relation to the upcoming competition” (Jones et al., 2005) to “how you usually feel before an important competition.”

#### 2.2.4. The Psychobiosocial Experience Semantic Differential scale in Sport

The Psychobiosocial Experience Semantic Differential scale in Sport (PESD-Sport; Robazza et al., 2021) comprises 30 bipolar items loading into 10 subscales (3 items each) to assess the psychological, bodily, and social modalities of psychobiosocial experiences. The psychological modality includes: emotion u/p (unpleasant/pleasant; e.g., “unhappy–happy”), confidence (e.g., “unconfident–confident”), anxiety (e.g., “worried in a harmful way–worried in a helpful way”), assertiveness (e.g., “submissive–fighting spirit”), and cognitive (e.g., “distracted–alert”) items. The bodily modality encompasses: bodily-somatic (e.g., “physically weak–physically vigorous”) and motor-behavioral (e.g., “uncoordinated in my movements–coordinated in my movements”) items. The social modality contains: operational (e.g., “ineffective in my performance–effective in my performance”), communicative (e.g., “being communicative is harmful–being communicative is useful”), and social support (e.g., “I feel ignored–I feel considered”) items. Each item is anchored by an adjective and its antonym in a semantic differential format. Dysfunctional adjectives for performance are on the left of a Likert-type scale while functional antonyms are on the right. Thinking about “how you usually feel before an important competition,” items are scored on a bipolar Likert-type scale ranging from 4 (*very much*) to 0 (*neither … nor*) on the “dysfunctional” side and from 0 to 4 on the “functional” side. Ratings on the dysfunctional side are then transformed into negative scores. Support for the factor structure and reliability was found in a sample of Italian athletes (Robazza et al., 2021), with *ω* values ranging from 0.740 (communicative) to 0.875 (social support).

### 2.3. Procedure

The study was carried out in accordance with the Declaration of Helsinki and was approved by the first author’s institutional ethics committee (No. 19, 09/09/2021). Participants were recruited by directly approaching sport club managers and head coaches, sending them a study information letter via email followed by telephone contact. After agreement to participate was granted, the general aim of the study and detailed procedures were presented in a meeting with sport managers and coaches before contacting the athletes. The criteria for participation in the study required that the athletes be currently active, practiced at least twice a week, have a minimum of 6 months experience of regular training in the sport, compete consistently during the sporting season, and be at least 16 years old. Before providing informed consent, the athletes were informed about the general objective of the study, the voluntary nature of participation, the possibility to withdraw from the study at any time without consequences, and the confidentiality of their responses. Informed parental consent was obtained for participants under the age of 18. The questionnaires were completed individually in a quiet room prior to a practice session, with an investigator administering the questionnaires in groups of no more than five participants.

### 2.4. Data analysis

After data screening for potential outliers, assumptions of normality, linearity, multicollinearity, and homoscedasticity (Hair et al., 2019), we examined the factorial validity of the measures through confirmatory factor analysis (CFA) performed in M*plus* 8.5 (Muthén and Muthén, 2017) using the maximum likelihood (MLR) parameter estimator with standard errors and a chi-square test statistic that are robust to non-normality. Model fit was assessed with comparative fit index (CFI), Tucker Lewis fit index (TLI), root mean square error of approximation (RMSEA), and standardized root mean square residual (SRMR). Adequate fit was inferred with values of CFI and TLI > 0.90, and RMSEA and SRMR lower than 0.08 (Whittaker and Schumacker, 2022). Good fit was assumed with CFI and TLI values close or higher to 0.95, and RMSEA and SRMR lower than 0.06 (Hu and Bentler, 1999).

McDonald’s *ω* values were computed to assess reliability of the measures. Convergence among a set of items representing a latent construct of the whole measurement model was examined through the average variance extracted (AVE) of the latent variables. AVE values close to or larger than 0.50 are deemed to support convergent validity of the measurement model (Hair et al., 2019). Furthermore, discriminant validity was established by comparing the AVE estimates of each factor with the squared interconstruct correlations related to that factor. Discriminant validity is assumed when AVE estimates are greater than the corresponding interconstruct squared correlation values (Hair et al., 2019).

Differences by gender and sport categories (i.e., individual vs. team) on the item mean scores of the dependent variables (i.e., the subscales of the measures) were evaluated through multivariate analysis of variance (MANOVA). Without previous evidence in support of an expected effect size for *a priori* power calculation, Kang and Jin (2016) recommended using a medium effect size with an alpha of 0.05 and an expected power of 0.80. The sample size was estimated using G*Power software (Version 3.1.9.7; Faul et al., 2009), with *f* = 0.25 (medium effect size), *β* = 0.80, and α = 0.05. The resulting recommended sample size was 330, so the initial sample size of 430 participants in our study was adequate.

Finally, structural equation modeling (SEM) was performed in M*plus* to test the indirect effects in the relationship between basic psychological needs and emotions/functional experiences via emotion regulation strategies. Mediation effects were tested using the maximum likelihood (ML) estimator and the bias-corrected bootstrap method based on 5,000 resamples with a 95% confidence interval around the standardized estimate (*β*). The sample size for SEM was established using the root mean square error of approximation (RMSEA; Myers et al., 2016). The minimum sample size for RMSEA was computed using the code developed by Preacher and Coffman (2006) for the R program (https://cran.r-project.org/). A sample size of 195 resulted after setting the type I error rate to α = 0.05, power = 0.80, null RMSE = 0.05, alternative RMSE = 0.04, and *df* = 729. Again, the sample size in the present study was adequate.

## 3. Results

### 3.1. Confirmatory factor analysis

Six cases were discarded because identified as univariate or multivariate outliers (Mahalanobis’ distance, *p* < 0.001). The final sample consisted of 424 participants. CFA on the BNSSS, ERQ, and SEQ data did not yield an acceptable fit, as reflected by poor loadings (< 0.40) of some items in the expected factor or cross-loadings. After the removal of problematic items, an acceptable fit to the data was obtained (Table 1) with values for comparative fit (CFI) and Tucker Lewis fit (TLI) indices >0.92, RMSEA and standardized root mean square residuals (SRMR) < 0.06 (Gunzler et al., 2021). McDonald’s ω reliability values ranged from 0.66 to 0.89. An acceptable fit to the data was also observed for the two measurement models relating the first to BNSSS, ERQ, and SEQ, and the second to BNSSS, ERQ, and PESD-Sport (Table 1). Acceptable convergent validity of the measurement model encompassing all measures was found, with most AVE values close to or above 0.50 (Table 1). In addition, adequate discriminant validity was observed after taking the lowest AVE value among the factors (i.e., 0.339 for Expressive suppression) as a reference. In fact, the AVE estimates were greater than the squared correlations between two latent factors for 160 of the 190 correlations.

**Table 1:** Confirmatory factor analysis fit indices and reliability values of the measures and measurement models.

Measure	Factor (number of items)	*χ*^2^/*df*	CFI	TLI	RMSEA (90% CI)	SRMR	*ω*	AVE
BNSSS		2.501	0.954	0.932	0.059 (0.041–0.079)	0.048		
	Competence (3)						0.659	0.400
	Autonomy-choice (3)						0.738	0.507
	Relatedness (3)						0.815	0.599
ERQ		1.403	0.981	0.973	0.031 (0.000–0.053)	0.035		
	Cognitive reappraisal (5)						0.741	0.371
	Expressive suppression (4)						0.667	0.339
SEQ		1.835	0.942	0.931	0.044 (0.036–0.052)	0.053		
	Anxiety (4)						0.808	0.506
	Dejection (5)						0.802	0.463
	Anger (3)						0.668	0.417
	Excitement (4)						0.805	0.504
	Happiness (4)						0.879	0.645
PESD-Sport		1.884	0.940	0.928	0.046 (0.040–0.051)	0.041		
	Emotion u/*p* (3)						0.854	0.661
	Confidence (3)						0.805	0.575
	Anxiety (3)						0.822	0.606
	Assertiveness (3)						0.786	0.549
	Cognitive (3)						0.836	0.642
	Bodily-somatic (3)						0.853	0.662
	Motor-behavioral (3)						0.815	0.583
	Operational (3)						0.831	0.629
	Communicative (3)						0.768	0.522
	Social support (3)						0.890	0.724
^1^BNSSS, ERQ, SEQ		1.475	0.934	0.925	0.033 (0.029–0.038)	0.051		
^1^BNSSS, ERQ, PESD-Sport		1.541	0.934	0.924	0.036 (0.032–0.039)	0.043		

BNSSS, Basic Needs Satisfaction in Sport Scale; ERQ, Emotion Regulation Questionnaire; SEQ, Sport Emotion Questionnaire; PESD-Sport, Psychobiosocial Experience Semantic Differential scale in Sport; *χ*^2^/*df*, chi-square/degrees of freedom; CFI, comparative fit index; TLI, Tucker Lewis fit index; RMSEA, root mean square error of approximation; SRMR, standardized root mean square residual; McDonald’s *ω*, omega values; AVE, average variance extracted. ^1^Measurement model.

### 3.2. Descriptive and inferential statistics

Descriptive statistics and correlation coefficients of latent variables are reported in Table 2. An inspection of correlation coefficients showed that (a) Competence was positively related to pleasant emotions (i.e., Excitement and Happiness), and psychobiosocial experiences, except for the Communicative modality, and negatively related to Expressive suppression and unpleasant emotions (i.e., Anxiety, Dejection, and Anger); (b) Autonomy was positively associated with Cognitive reappraisal; (c) Relatedness was positively linked to Cognitive reappraisal, pleasant emotions, and psychobiosocial experiences, except for Anxiety and Communicative modalities, and negatively linked to Expressive suppression, Dejection, and Anger; (d) Cognitive reappraisal was positively associated with pleasant emotions and Psychobiosocial Experiences, except for the Communicative modality; and (e) Expressive suppression was positively associated with Dejection, Anger, and the Communicative modality of psychobiosocial experiences, and negatively associated with Emotion u/p. All correlations were in the expected direction, except for Expressive suppression, which was significantly correlated with the Communicative modality of psychobiosocial experiences.

**Table 2:** Descriptive statistics for women and men involved in individual and team sports, and correlation coefficients of latent variables for the whole sample (*N* = 424).

	Women		Men																				
	Individual (*n* = 75)	Team (*n* = 103)	Individual (*n* = 89)	Team (*n* = 157)																			
Variable	*M* ± *SD*	*M* ± *SD*	*M* ± *SD*	*M* ± *SD*	1	2	3	4	5	6	7	8	9	10	11	12	13	14	15	16	17	18	19
Basic psychological needs																							
1. Competence	4.95 ± 1.10	4.98 ± 1.38	5.05 ± 1.39	4.94 ± 1.35	––																		
2. Autonomy	4.85 ± 1.42	3.53 ± 1.37	4.92 ± 1.55	3.98 ± 1.33	0.05	––																	
3. Relatedness	5.53 ± 1.17	5.59 ± 1.28	5.90 ± 1.15	5.27 ± 1.28	0.21^*^	0.16	––																
Emotion regulation																							
4. Cognitive reappraisal	2.53 ± 0.71	2.54 ± 0.61	2.74 ± 0.62	2.69 ± 0.52	0.17	0.27^*^	0.22^*^	––															
5. Expressive suppression	2.09 ± 0.63	2.22 ± 0.75	2.27 ± 0.63	2.49 ± 0.62	−0.25^*^	−0.05	−0.23^*^	0.03	––														
Sport emotions																							
6. Anxiety	2.13 ± 0.97	1.66 ± 0.81	1.34 ± 0.80	1.48 ± 0.83	−0.22^*^	−0.05	−0.03	−0.15	0.07	––													
7. Dejection	0.36 ± 0.53	0.29 ± 0.42	0.20 ± 0.40	0.53 ± 0.68	−0.45^§^	−0.04	−0.29^*^	−0.14	0.24^*^	0.32^*^	––												
8. Anger	0.40 ± 0.68	0.51 ± 0.69	0.37 ± 0.61	0.76 ± 0.83	−0.33^*^	−0.09	−0.27^*^	−0.04	0.20^*^	0.36^*^	0.79^†^	––											
9. Excitement	2.59 ± 0.89	2.81 ± 0.73	2.59 ± 0.84	2.73 ± 0.88	0.25^*^	0.10	0.27^*^	0.38^*^	−0.16	0.08	−0.31^*^	−0.08	––										
10. Happiness	2.56 ± 0.96	2.92 ± 0.81	2.69 ± 0.87	2.74 ± 0.95	0.26^*^	0.08	0.33^*^	0.36^*^	−0.16	−0.16	−0.31^*^	−0.24^*^	0.90^#^	––									
Psychobiosocial experiences																							
11. Emotion u/p	2.29 ± 1.32	2.76 ± 1.15	2.74 ± 1.31	2.56 ± 1.43	0.36^*^	0.13	0.42^§^	0.39^*^	−0.24^*^	−0.18	−0.43^§^	−0.30^*^	0.60^†^	0.68^†^	––								
12. Confidence	1.36 ± 1.59	2.05 ± 1.34	2.26 ± 1.21	2.27 ± 1.41	0.43^§^	0.11	0.33^*^	0.39^*^	−0.15	−0.41^§^	−0.36^*^	−0.13	0.53^§^	0.55^§^	0.81^#^	––							
13. Anxiety	0.87 ± 1.68	1.11 ± 1.62	1.30 ± 1.55	1.52 ± 1.46	0.32^*^	0.16	0.09	0.37^*^	−0.12	−0.25^*^	−0.18	−0.02	0.39^*^	0.37^*^	0.55^§^	0.73^†^	––						
14. Assertiveness	2.10 ± 1.46	2.67 ± 1.16	2.66 ± 0.91	2.59 ± 1.36	0.35^*^	0.04	0.25^*^	0.35^*^	−0.13	−0.13	−0.30^*^	−0.06	0.64^†^	0.48^§^	0.73^†^	0.80^#^	0.63^†^	––					
15. Cognitive	2.43 ± 1.24	2.54 ± 1.15	2.69 ± 1.21	2.49 ± 1.42	0.25^*^	0.11	0.20^*^	0.22^*^	0.00	−0.13	−0.23^*^	−0.21^*^	0.38^*^	0.31^*^	0.56^§^	0.61^†^	0.51^§^	0.68^†^	––				
16. Bodily-somatic	1.95 ± 1.59	2.36 ± 1.18	2.59 ± 1.19	2.38 ± 1.49	0.26^*^	0.06	0.21^*^	0.33^*^	0.02	−0.13	−0.19	−0.05	0.55^§^	0.45^§^	0.71^†^	0.70^†^	0.52^§^	0.79^†^	0.60^†^	––			
17. Motor-behavioral	2.15 ± 1.29	2.09 ± 1.26	2.54 ± 1.04	2.46 ± 1.29	0.28^*^	0.08	0.21^*^	0.31^*^	−0.10	−0.15	−0.25^*^	−0.15	0.45^§^	0.34^*^	0.58^§^	0.69^†^	0.56^§^	0.67^†^	0.74^†^	0.81^#^	––		
18. Operational	1.84 ± 1.42	2.05 ± 1.16	2.23 ± 1.25	2.27 ± 1.33	0.35^*^	0.09	0.23^*^	0.32^*^	−0.10	−0.20^*^	−0.24^*^	−0.12	0.53^§^	0.48^§^	0.68^†^	0.83^#^	0.70^†^	0.71^†^	0.72^†^	0.79^†^	0.92^#^	––	
19. Communicative	0.44 ± 1.44	0.08 ± 1.82	0.58 ± 1.61	0.82 ± 1.68	−0.08	0.05	−0.19	−0.04	0.46^§^	−0.05	0.17	0.20^*^	−0.08	−0.03	0.01	0.17	0.28^*^	0.09	0.24^*^	0.20^*^	0.23^*^	0.16	––
20. Social support	2.20 ± 1.40	2.43 ± 1.47	2.62 ± 1.11	2.24 ± 1.56	0.33^*^	0.17	0.48^§^	0.31^*^	−0.19	−0.14	−0.35^*^	−0.22^*^	0.46^§^	0.47^§^	0.83^#^	0.68^†^	0.45^§^	0.53^§^	0.57^§^	0.54^§^	0.53^§^	0.61^†^	0.03

Correlation ^*^low, ^§^moderate, ^†^moderately high ^#^high (Zhu, 2012).

MANOVA yielded significant differences by gender, sport type, and gender by sport type interaction. The complete results are presented in the Supplementary materials, and follow-up comparisons are reported in Supplementary Table 2. To account for these differences, gender, sport type, and gender by sport type interaction were entered as covariates into subsequent SEM analyses.

### 3.3. Structural equation modeling

#### 3.3.1. Basic psychological needs, emotion regulation, and emotions

**Figure 1:**
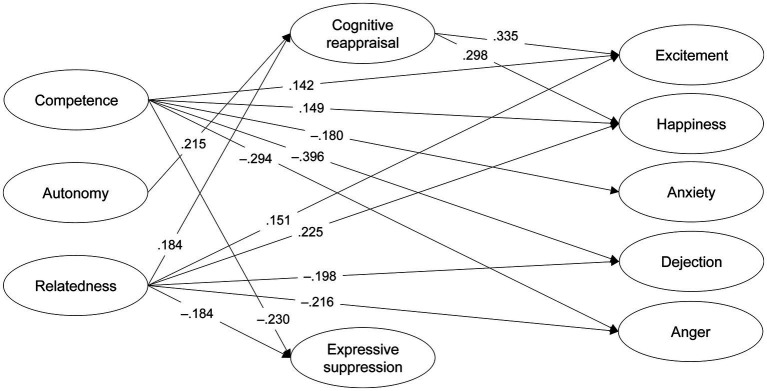
Structural equation model illustrating the relationships between basic needs (competence, autonomy, and relatedness), emotion regulation strategies (cognitive reappraisal and expressive suppression), and emotions, controlling for gender, sport type, and gender by sport type interaction (covariates not shown for the sake of clarity). Only significant standardized estimates are presented (*p* <  0.05).

SEM results regarding the relationships between basic needs satisfaction, emotion regulation strategies, and sport emotions showed that Competence was positively related to Excitement and Happiness, and negatively linked to Expressive suppression, Anxiety, Dejection, and Anger. Autonomy and Relatedness were positively related to Cognitive reappraisal. Relatedness was also positively associated with Excitement and Happiness, and negatively linked to Expressive suppression, Dejection, and Anger (Table 3 and Figure 1). Furthermore, Cognitive reappraisal was positively linked to Excitement and Happiness. Mediation analysis showed positive indirect effects from Autonomy and Relatedness to Excitement and Happiness via Cognitive reappraisal (Supplementary Table 3).

**Table 3:** Standardized estimates and 95% confidence intervals from structural equation modeling results of the relationships between basic needs (competence, autonomy, and relatedness), emotion regulation strategies (cognitive reappraisal and expressive suppression), and emotions.

Relationship	Lower 2.5%	Estimate	Upper 2.5%
Competence → Cognitive reappraisal	−0.004	0.128	0.259
Autonomy → Cognitive reappraisal	0.086	0.215*	0.344
Relatedness → Cognitive reappraisal	0.068	0.184*	0.299

Competence → Expressive suppression	−0.376	−0.230*	−0.084
Autonomy → Expressive suppression	−0.179	−0.047	0.084
Relatedness → Expressive suppression	−0.309	−0.184*	−0.060

Competence → Excitement	0.023	0.142*	0.262
Autonomy → Excitement	−0.144	−0.034	0.077
Relatedness → Excitement	0.025	0.151*	0.276
Cognitive reappraisal → Excitement	0.220	0.335*	0.450
Expressive suppression → Excitement	−0.248	−0.112	0.024

Competence → Happiness	0.026	0.149*	0.271
Autonomy → Happiness	−0.159	−0.055	0.049
Relatedness → Happiness	0.104	0.225*	0.345
Cognitive reappraisal → Happiness	0.183	0.298*	0.413
Expressive suppression → Happiness	−0.225	−0.096	0.033

Competence → Anxiety	−0.317	−0.180*	−0.043
Autonomy → Anxiety	−0.112	0.014	0.140
Relatedness → Anxiety	−0.111	0.019	0.149
Cognitive reappraisal → Anxiety	−0.218	−0.084	0.049
Expressive suppression → Anxiety	−0.064	0.081	0.226

Competence → Dejection	−0.510	−0.396*	−0.282
Autonomy → Dejection	−0.099	0.005	0.110
Relatedness → Dejection	−0.327	−0.198*	−0.068
Cognitive reappraisal → Dejection	−0.183	−0.052	0.079
Expressive suppression → Dejection	−0.067	0.078	0.222

Competence → Anger	−0.449	−0.294*	−0.139
Autonomy → Anger	−0.163	−0.015	0.133
Relatedness → Anger	−0.364	−0.216*	−0.069
Cognitive reappraisal → Anger	−0.078	0.062	0.202
Expressive suppression → Anger	−0.091	0.063	0.217

*p < 0.05.

#### 3.3.2. Basic psychological needs, emotion regulation, and psychobiosocial experiences

**Figure 2:**
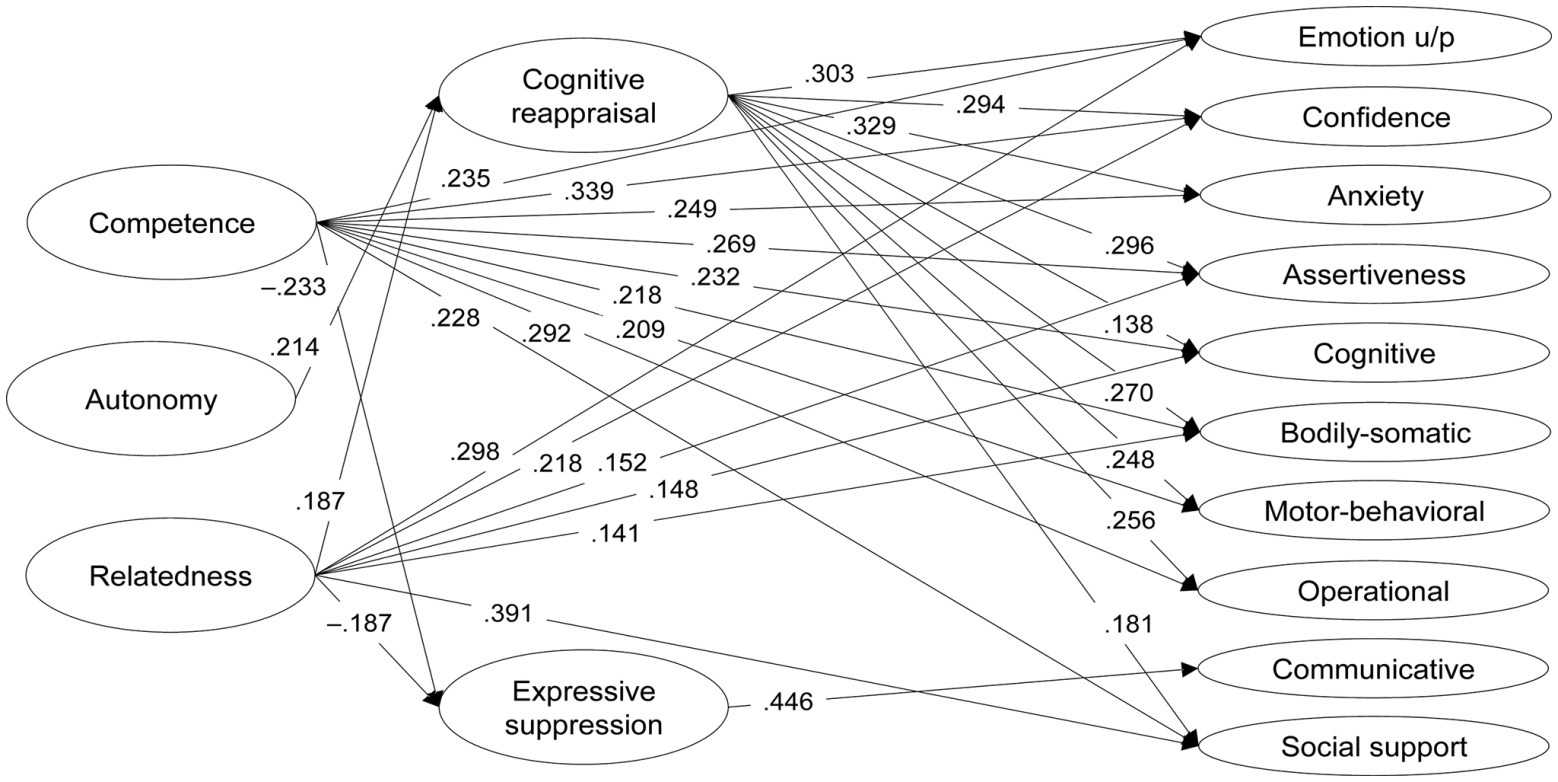
Structural equation model illustrating the relationships between basic needs (competence, autonomy, and relatedness), emotion regulation strategies (cognitive reappraisal and expressive suppression), and modalities of psychobiosocial experiences, controlling for gender, sport type, and gender by sport type interaction (covariates not shown for the sake of clarity). Only significant standardized estimates are presented (*p* <  0.05).

SEM results on the relationships between basic needs satisfaction, emotion regulation strategies, and the modalities of psychobiosocial experiences showed that Competence and Relatedness were positively linked to most modalities of psychobiosocial experiences. Additionally, Autonomy and Relatedness were positively associated with Cognitive reappraisal, while Competence was negatively related to Expressive suppression. Cognitive reappraisal was positively linked to all modalities of psychobiosocial experiences except for the Communicative modality which was positively linked to Expressive suppression (Table 4 and Figure 2).

**Table 4:** Standardized estimates and 95% confidence intervals from structural equation model results of the relationships between basic needs (competence, autonomy, and relatedness), emotion regulation strategies (cognitive reappraisal and expressive suppression), and psychobiosocial experiences.

Relationship	Lower 2.5%	Estimate	Upper 2.5%
Competence → Cognitive reappraisal	−0.015	0.120	0.255
Autonomy → Cognitive reappraisal	0.085	0.214*	0.343
Relatedness → Cognitive reappraisal	0.071	0.187*	0.302

Competence → Expressive suppression	−0.381	−0.233*	−0.086
Autonomy → Expressive suppression	−0.177	−0.046	0.084
Relatedness → Expressive suppression	−0.311	−0.187*	−0.064

Competence → Emotion u/p	0.122	0.235*	0.348
Autonomy → Emotion u/p	−0.130	−0.020	0.090
Relatedness → Emotion u/p	0.176	0.298*	0.420
Cognitive reappraisal → Emotion u/p	0.192	0.303*	0.415
Expressive suppression → Emotion u/p	−0.257	−0.129	−0.001

Competence → Confidence	0.212	0.339*	0.466
Autonomy → Confidence	−0.150	−0.035	0.079
Relatedness → Confidence	0.094	0.218*	0.342
Cognitive reappraisal → Confidence	0.176	0.294*	0.411
Expressive suppression → Confidence	−0.175	−0.053	0.069

Competence → Anxiety	0.122	0.249*	0.376
Autonomy → Anxiety	−0.075	0.048	0.171
Relatedness → Anxiety	−0.165	−0.045	0.075
Cognitive reappraisal → Anxiety	0.206	0.329*	0.452
Expressive suppression → Anxiety	−0.222	−0.086	0.050

Competence → Assertiveness	0.144	0.269*	0.394
Autonomy → Assertiveness	−0.206	−0.084	0.038
Relatedness → Assertiveness	0.018	0.152*	0.285
Cognitive reappraisal → Assertiveness	0.180	0.296*	0.412
Expressive suppression → Assertiveness	−0.167	−0.038	0.091

Competence → Cognitive	0.111	0.232*	0.353
Autonomy → Cognitive	−0.075	0.049	0.173
Relatedness → Cognitive	0.022	0.148*	0.273
Cognitive reappraisal → Cognitive	0.022	0.138*	0.254
Expressive suppression → Cognitive	−0.039	0.088	0.215

Competence → Bodily-somatic	0.107	0.218*	0.329
Autonomy → Bodily-somatic	−0.142	−0.035	0.073
Relatedness → Bodily-somatic	0.023	0.141*	0.260
Cognitive reappraisal → Bodily-somatic	0.163	0.270*	0.378
Expressive suppression → Bodily-somatic	−0.026	0.094	0.215
Competence → Motor-behavioral	0.074	0.209*	0.344
Autonomy → Motor-behavioral	−0.148	−0.020	0.108
Relatedness → Motor-behavioral	−0.016	0.119	0.254
Cognitive reappraisal → Motor-behavioral	0.125	0.248*	0.371
Expressive suppression → Motor-behavioral	−0.171	−0.039	0.092

Competence → Operational	0.162	0.292*	0.422
Autonomy → Operational	−0.137	−0.015	0.106
Relatedness → Operational	−0.010	0.128	0.265
Cognitive reappraisal → Operational	0.130	0.256*	0.381
Expressive suppression → Operational	−0.124	0.002	0.128

Competence → Communicative	−0.076	0.058	0.192
Autonomy → Communicative	−0.015	0.101	0.218
Relatedness → Communicative	−0.205	−0.082	0.040
Cognitive reappraisal → Communicative	−0.225	−0.088	0.049
Expressive suppression → Communicative	0.299	0.446*	0.593

Competence → Social support	0.107	0.228*	0.349
Autonomy → Social support	−0.049	0.057	0.163
Relatedness → Social support	0.270	0.391*	0.511
Cognitive reappraisal → Social support	0.065	0.181*	0.298
Expressive suppression → Social support	−0.176	−0.054	0.068

*p < 0.05.

Mediation analysis revealed positive indirect effects through Cognitive reappraisal from Autonomy and Relatedness to most modalities: Emotion u/p, Confidence, Anxiety, Assertiveness, Cognitive, Bodily-somatic, Motor-behavioral, Operational, and Social support. Moreover, negative indirect effects through expressive suppression were observed from Competence and Relatedness to the Communicative modality (Supplementary Table 4).

## 4. Discussion

In this study, we investigated the role of athletes’ emotion regulation strategies in the relationship between basic psychological needs satisfaction, emotions, and functional psychobiosocial experiences, following the tenets of the basic psychological needs theory (Ryan and Deci, 2017), the process model of emotion regulation (Gross, 1998, 2014), and the IZOF model conceptualization of psychobiosocial states (Hanin, 2007). Taken as a whole, the findings concur with and extend those of a previous study framed within the achievement goal theory (Nicholls, 1984), in which the perceived motivational climate (i.e., mastery and performance) created by the coach was antecedent of emotion regulation strategies and emotions/functional experiences (Robazza et al., 2022).

### 4.1. Hypothesis 1: basic psychological needs → emotion regulation, and emotions/psychobiosocial experiences

Hypothesis 1 was partially supported, with autonomy and relatedness need satisfaction (but not competence) positively related to cognitive reappraisal, and competence and relatedness need satisfaction (but not autonomy) negatively linked to expressive suppression. Moreover, competence and relatedness were associated positively with pleasant emotions and negatively with unpleasant emotions. Competence and relatedness were positively related to most modalities of psychobiosocial experiences, likely due to the positive mean scores observed on all modalities across gender and sport type. Positive scores on all modalities had previously been observed in two samples of athletes (Robazza et al., 2021), indicating that the PESD-Sport mainly assesses functional experiences.

The results concur with previous research indicating that the satisfaction of athletes’ basic psychological needs was positively associated with enjoyment (e.g., Jaakkola et al., 2016), optimal social functioning, well-being, and self-development (e.g., Cheval et al., 2017), and negatively related to burnout and ill-being (e.g., Balaguer et al., 2012; for a review, see Raabe et al., 2019). Findings also complement those of Robazza et al. (2022), who found that perceived mastery climate was positively associated with cognitive reappraisal and pleasant emotions/functional experiences. The results of the previous and the present study taken together suggest that a mastery motivational climate, as conceived within goal achievement theory, and the satisfaction of basic psychological needs, as conceptualized within self-determination theory, are associated with adaptive emotions and emotion-related experiences. In this regard, Duda (2013) combined the theoretical notions and applied indications stemming from the two theoretical perspectives within the so-called “Empowering Coaching” program, which aims to help coaches create a more empowering motivational climate, assumed to satisfy athletes’ psychological needs and promote their quality of engagement in sport and overall health (Duda and Appleton, 2016; for a review, see Birr et al., 2023). In a sample of British athletes, Ruiz et al. (2021a) reported direct and indirect effects of an empowering climate to happiness and excitement via autonomous motivation, and of a disempowering climate to dejection and anger via controlled motivation. The results from the current study are in line with this hierarchical conceptualization of the motivational climate and previous research findings.

### 4.2. Hypothesis 2: emotion regulation → emotions/psychobiosocial experiences

Hypothesis 2 was also partially supported, with cognitive reappraisal being positively linked to pleasant emotions and most psychobiosocial experiences. The results align with research findings from the general population of predominantly Western cultural background (e.g., Gross and John, 2003; Preece et al., 2020) and with athletic samples (Cece et al., 2019), indicating that the antecedent-focused strategy of cognitive reappraisal is usually associated with pleasant affect (e.g., Balzarotti et al., 2010; Ioannidis and Siegling, 2015). Interestingly, the correlation between the cognitive reappraisal and expressive suppression scores was close to zero, suggesting that these are two independent regulatory strategies (John and Eng, 2014).

The pattern of correlations between expressive suppression and emotions was as expected (i.e., negative with pleasant emotions and positive with unpleasant emotions), although the only significant correlations were found with dejection and anger. Regarding psychobiosocial experiences, expressive suppression correlated negatively with most modalities as predicted, although the only significant correlations were observed with the emotion u/p and communicative modalities. Interestingly, the correlation with the communicative modality was positive, as also found in Robazza et al.’s (2022) study, indicating that communication with significant others (e.g., coaches and peers) may be facilitated when the athletes’ externalization of unpleasant experiences is inhibited.

Finally, the lack of a significant correlation between scores of expressive suppression and anxiety may be interpreted in light of research evidence showing that anxiety symptoms can be appraised not only as debilitative, but also as facilitative, depending on the individual’s perceived impact on performance (Jones et al., 1994; Neil et al., 2012). Indeed, symptoms such as increased heart rate and muscle tension during competition, while unpleasant, may be perceived by the athlete as helpful in energizing their behavior and keeping their attention focused on the task. Therefore, athletes who appraise their anxiety symptoms as helpful may not need to suppress them.

### 4.3. Hypothesis 3: basic psychological needs → emotion regulation → emotions/psychobiosocial experiences

Regarding Hypothesis 3, findings support the expected indirect effects of emotion regulation strategies on the relationship between autonomy and relatedness needs satisfaction and pleasant emotions (i.e., happiness and excitement), as well as most modalities of psychobiosocial experiences via cognitive reappraisal. These findings align with and extend those of an earlier study, which showed that perceived mastery climate had positive indirect effects on psychobiosocial experiences through cognitive reappraisal (Robazza et al., 2022). This is as one would expect, considering that in a mastery climate, the coach’s attention is on individual criteria of success and positive interactions with peers, rather than on external criteria of success and outperforming others. The results of the current study, as well as existing empirical evidence, support the view that a coach-created empowering motivational climate (Duda and Appleton, 2016), characterized by the satisfaction of individual basic needs of competence, autonomy, and relatedness (Ryan and Deci, 2017) in a mastery climate (Nicholls, 1984), is accompanied by adaptive emotion regulation (i.e., cognitive reappraisal), pleasant emotions, and functional psychobiosocial experiences. In addition, negative indirect effects emerged from competence and relatedness to the communicative modality of psychobiosocial experiences through expressive suppression. As previously noted, this may be due to the positive correlation between expressive suppression and the communicative modality, suggesting that communication may be improved when the athletes inhibit their display of unpleasant feelings.

### 4.4. Gender differences

Lastly, gender differences are worth noting. In particular, men reported higher scores on both emotion regulation strategies, confidence, and functional anxiety, and lower scores on unpleasant anxiety than women. Moreover, women involved in individual sports scored higher on unpleasant anxiety. These differences are likely due to gender distinctions created by stereotypes and norms embedded in the social and sport systems. These social influences can impact how emotions and related feelings are expressed and, consequently, the use of emotion regulation strategies (Morano et al., 2020b; for a review, see Gill, 2020).

### 4.5. Practical implications

From an applied standpoint, coaches should provide athletes with a supportive environment to enhance their sense of competence, autonomy, and relatedness (Greenlees, 2022), and promote the experience of pleasant emotions and functional feeling states. Schüler et al. (2023) offered several suggestions on how to promote satisfaction of basic psychological needs in sport. Coaches can foster autonomy by providing athletes with opportunities to make decisions about their sport participation in training and competition, allowing them to express their opinions and preferences, and assisting them in making decisions that are consistent with their goals and values. Competence can be improved by providing informative feedback focused on improvements, setting realistic and achievable goals, and designing practice and competition environments that match the individual’s skill levels and abilities. The sense of relatedness can be strengthened by providing opportunities for social interaction and promoting a supportive and inclusive environment in which all athletes feel valued and included.

Furthermore, practitioners should help athletes adopt an adaptive emotion regulation style, focused on cognitive reappraisal rather than engaging in expressive suppression, to improve their sporting experience and well-being. Athletes should be informed about the differences between adaptive and maladaptive emotion regulation strategies, and the advantages of cognitive reappraisal over expressive suppression in terms of emotional responses and performance outcomes (Uphill et al., 2009). Practitioners should promote open communication in which athletes feel comfortable expressing their feelings and are willing to identify, reframe, and cognitively reappraise dysfunctional thoughts and emotions in training and competition (Lane et al., 2012). Examples of athletes’ dysfunctional appraisal are, “I am feeling nervous about this event. I am afraid of embarrassing myself in front of everyone,” and “I have already failed my goal under pressure. I did it at a decisive moment in the competition.” Suggested adaptive alternatives can be, “Feeling nervous is normal before an event. I can use this energy to focus on my goals and give my best,” and “Yes, I feel the pressure, but I have learned from my previous mistakes. I have worked hard and have the skills to deal with it. I just need to stay focused and trust my abilities.”

### 4.6. Limitations and future directions

The cross-sectional nature of this investigation does not allow to establish causal relationships between variables, which also limits the generalizability of the findings. To determine causality, longitudinal or experimental studies are needed to assess the effect of one variable on other variables over time or as a result of an intervention.

Another study limitation is its focus on basic psychological needs and cognitive reappraisal, which represent narrowed aspects of athletes’ motivation and emotion regulation within the broader frameworks of the self-determination theory (Ryan and Vansteenkiste, 2023) and the process model of emotion regulation (Gross, 1998, 2014, 2015). While basic psychological needs and cognitive reappraisals are important, they do not cover all factors that motivate and regulate goal-directed behavior. Therefore, a wider approach should consider, for example, individual differences, the dynamics of intrinsic and extrinsic motivations, the roles of expectancies and goals, and the environmental and social factors that influence motivation (Ryan, 2019), as well as a range of emotion regulation strategies used by different individuals (English et al., 2021). This approach could provide a more comprehensive understanding of the interplay between athletes’ motivational factors, emotion regulation, and emotional responses on performance processes and outcomes.

Finally, we examined gender and sport type differences in the studied variable scores. Possible differences by age, experience, and competitive level could not be examined due to the unequal distribution of these categories in the sample. Future studies should involve a more balanced number of participants in terms of age, experience, competitive level, gender, and sport type, as well as establish measurement and structural invariance of the measures.

## 5. Conclusion

Findings suggest a positive relationship between athletes’ basic psychological needs satisfaction and the use of cognitive reappraisal (i.e., an adaptive emotion regulation strategy), which involves changing the way a situation is evaluated in the sport context to regulate one’s emotions. This, in turn, can lead athletes to experience pleasant emotions and a range of functional psychobiosocial experiences. The results are consistent with the tenets of basic psychological needs theory, within the broader perspective of self-determination theory, which proposes that satisfaction of psychological needs for autonomy, competence, and relatedness is essential for optimal motivation, engagement, and well-being. Overall, these findings suggest that promoting the satisfaction of basic psychological needs in athletes may have important implications for their emotion regulation. Coaches and practitioners can use this information to design interventions that promote basic psychological needs satisfaction and encourage the use of adaptive emotion regulation strategies. Further research is needed to determine the final impact of basic psychological needs, emotion regulation styles, and emotion-related experiences on athletes’ performance and well-being.

## Data availability statement

The raw data supporting the conclusions of this article will be made available by the authors, without undue reservation.

## Ethics statement

The studies involving human participants were reviewed and approved by “G. d’Annunzio” University of Chieti-Pescara. Written informed consent to participate in this study was provided by the participants or their legal guardian/next of kin.

## Author contributions

CR and LB collected the data. CR performed the statistical analysis. All authors conceived the study, interpreted the results of the research, contributed to manuscript writing and revision, and approved the submitted version.

## Conflict of interest

The authors declare that the research was conducted in the absence of any commercial or financial relationships that could be construed as a potential conflict of interest.

## Publisher’s note

All claims expressed in this article are solely those of the authors and do not necessarily represent those of their affiliated organizations, or those of the publisher, the editors and the reviewers. Any product that may be evaluated in this article, or claim that may be made by its manufacturer, is not guaranteed or endorsed by the publisher.
