# Supplementary material for: Athletes’ basic psychological needs and emotions: the role of cognitive reappraisal
Source: Front Psychol. 2023 Jul 13;14:1205102. doi: 10.3389/fpsyg.2023.1205102 (PMC10374325; doi:10.3389/fpsyg.2023.1205102)
Supplement: Supplementary file 1 [file Table_1.pdf]

Supplementary Table 1
Characteristics of the Participants

	Gender	
Age, Competitive experience, Level, Sport	Woman (*n* = 178)	Man (*n* = 246)
Age (Mean, *SD*)	22.07 yrs. (7.45)	24.09 yrs. (8.03)
Competitive experience (Mean, *SD*)	8.95 yrs. (5.92)	10.46 yrs. (6.95)
Competitive level (%)		
Regional	71.50	70.50
National	19.10	16.90
International	9.40	12.60
Sport (%)		
Archery	5.50	6.00
Baseball	–	1.80
Basketball	8.50	15.00
Cycling	.40	–
Dancesport	7.10	1.10
Fencing	1.60	2.18
Futsal	2.00	3.70
Handball	–	.60
Gymnastics	4.20	–
Martial arts	3.10	4.00
Motocross		.30
Rhythmic gymnastics	5.80	–
Rugby	–	3.40
Running	–	2.30
Shooting	1.60	5.20
Soccer	15.10	21.16
Softball	6.97	–
Swimming	2.00	7.10
Synchronized swimming	1.20	–
Track & field	6.93	8.00
Volleyball	25.30	15.36
Water polo	–	2.80
Windsurfing	2.70	–

Differences by Gender and Sport

Differences by gender and sport categories (i.e., individual vs. team) on the item mean scores of the dependent variables (i.e., the subscales of the measures) were evaluated through multivariate analysis of variance (MANOVA). MANOVA indicated significant differences by gender, Wilks' λ = .829, $F(20, 401) = 4.122$, $p < .001$, $\eta_p^2 = .171$, sport category, Wilks' λ = .736, $F(20, 401) = 7.199$, $p < .001$, $\eta_p^2 = .264$, and gender by sport interaction, Wilks' λ = .897, $F(20, 401) = 2.312$, $p = .001$, $\eta_p^2 = .103$. Follow-up comparisons (Supplementary Table 2) showed that men reported higher mean rating scores on Cognitive reappraisal, Expressive suppression, as well as the Confidence, Anxiety, Motor-behavioral, and Communicative modalities of psychobiosocial experiences, and lower scores in Anxiety. Compared to team sport athletes, ratings of individual sport athletes were higher in Autonomy and lower in Anger. Gender by Sport type interaction was significant for Relatedness, Anxiety, and Dejection. Post hoc analysis with Bonferroni adjustment showed that male athletes involved in individual sports reported higher scores on Relatedness than male athletes practicing team sports. Moreover, female athletes involved in individual sports reported higher scores on Anxiety, while male athletes practicing team sports scored higher on Dejection.

Supplementary Table 2

Gender, Sport, and Gender by Sport Univariate Follow-up Comparisons

	Gender			*Sport*			*Gender by Sport*		
Variable	$F(1, 420)$	p	η_p^2	$F(1, 420)$	p	η_p^2	$F(1, 420)$	p	η_p^2
Basic Psychological Needs									
Competence	0.035	.852	.000	0.089	.766	.000	0.294	.588	.001
Autonomy	3.312	.070	.008	63.782	<.001	.132	1.793	.181	.004
Relatedness	0.038	.846	.000	5.264	.022	.012	7.515	.006	.018
Emotion Regulation									
Cognitive reappraisal	8.721	.003	.020	0.099	.753	.000	0.281	.597	.001
Expressive suppression	11.659	.001	.027	6.761	.010	.016	0.377	.540	.001
Sport Emotions									
Anxiety	32.179	<.001	.071	3.643	.057	.009	13.206	<.001	.030
Dejection	0.470	.493	.001	5.498	.019	.013	13.497	<.001	.031
Anger	2.307	.130	.005	11.809	.001	.027	3.635	.057	.009
Excitement	0.267	.606	.001	4.519	.034	.011	0.226	.635	.001
Happiness	0.110	.741	.000	5.261	.022	.012	3.036	.082	.007
Psychobiosocial Experiences									
Emotion u/p	0.908	.341	.002	1.147	.285	.003	5.985	.015	.014
Confidence	16.217	<.001	.037	6.255	.013	.015	5.844	.016	.014
Anxiety	7.122	.008	.017	2.139	.144	.005	0.007	.931	.000
Assertiveness	3.577	.059	.008	3.890	.049	.009	6.591	.011	.015
Cognitive	0.603	.438	.001	0.101	.751	.000	1.410	.236	.003
Bodily-somatic	5.592	.018	.013	0.515	.473	.001	5.148	.024	.012
Motor-behavioral	9.168	.003	.021	0.329	.566	.001	0.004	.951	.000
Operational	5.565	.019	.013	0.953	.330	.002	0.393	.531	.001
Communicative	6.904	.009	.016	0.140	.708	.000	3.199	.074	.008
Social support	0.681	.410	.002	0.252	.616	.001	4.468	.035	.011

Supplementary Table 3

Results for Total, Total Indirect, Indirect, and Direct Effects for Paths from Antecedent Variables (i.e., Autonomy, and Relatedness) to Pleasant Emotions via Cognitive Reappraisal and Expressive Suppression

Effect	*β*	SE	Bootstrap Bias–Corrected 95% CI (Lower, Upper)	
Autonomy to Excitement				
Total	.044	.057	–.071	.153
Total indirect	.077*	.028	.032	.144
Autonomy → Reappraisal → Excitement	.072*	.026	.029	.132
Autonomy → Suppression → Excitement	.005	.010	–.006	.037
Autonomy → Excitement	–.034	.058	–.150	.074
Relatedness to Excitement				
Total	.233*	.064	.109	.358
Total indirect	.082*	.028	.036	.146
Relatedness → Reappraisal → Excitement	.061*	.022	.024	.112
Relatedness → Suppression → Excitement	.021	.017	–.002	.066
Relatedness → Excitement	.151*	.066	.027	.282
Autonomy to Happiness				
Total	.014	.054	–.094	.120
Total indirect	.069*	.026	.026	.130
Autonomy → Reappraisal → Happiness	.064*	.025	.024	.121
Autonomy → Suppression → Happiness	.005	.009	–.005	.033
Autonomy → Happiness	–.055	.054	–.160	.052
Relatedness to Happiness				
Total	.297*	.060	.170	.410
Total indirect	.072*	.026	.030	.134
Relatedness → Reappraisal → Happiness	.055*	.020	.022	.103
Relatedness → Suppression → Happiness	.018	.015	–.002	.060
Relatedness → Happiness	.225*	.063	.095	.346

Note. Only results for significant basic needs–emotion paths are included. *Significance indicated via 95% CI. Abbreviations: *β* = standardized estimate; SE = Standard error; CI = Confidence interval.

Supplementary Table 4

Significant Results for Total, Total Indirect, Indirect, and Direct Effects for Paths from Antecedent Variables (i.e., Competence, Autonomy, and Relatedness) to the Modalities of Psychobiosocial Experiences via Cognitive Reappraisal and Expressive Suppression

Effect	*β*	SE	Bootstrap Bias–Corrected 95% CI (Lower, Upper)	
Autonomy to Emotion u/p				
Total	.051	.056	–.057	.164
Total indirect	.071*	.026	.028	.133
Autonomy → Reappraisal → Emotion u/p	.065*	.024	.025	.123
Autonomy → Suppression → Emotion u/p	.006	.010	–.007	.037
Autonomy → Emotion u/p	–.020	.057	–.132	.095
Relatedness to Emotion u/p				
Total	.379*	.063	.248	.496
Total indirect	.081*	.025	.040	.141
Relatedness → Reappraisal → Emotion u/p	.057*	.019	.024	.102
Relatedness → Suppression → Emotion u/p	.024	.016	.000	.067
Relatedness → Emotion u/p	.298*	.064	.169	.417
Autonomy to Confidence				
Total	.030	.059	–.083	.152
Total indirect	.065*	.025	.025	.126
Autonomy → Reappraisal → Confidence	.063*	.024	.024	.119
Autonomy → Suppression → Confidence	.002	.006	–.004	.027
Autonomy → Confidence	–.035	.060	–.149	.087
Relatedness to Confidence				
Total	.283*	.060	.159	.400
Total indirect	.065*	.024	.024	.119
Relatedness → Reappraisal → Confidence	.055*	.020	.021	.100
Relatedness → Suppression → Confidence	.010	.013	–.011	.043
Relatedness → Confidence	.218*	.064	.087	.344
Autonomy to Anxiety				
Total	.122	.065	–.002	.252
Total indirect	.074*	.027	.029	.136
Autonomy → Reappraisal → Anxiety	.070*	.026	.028	.131
Autonomy → Suppression → Anxiety	.004	.008	–.005	.032
Autonomy → Anxiety	.048	.065	–.075	.181
Relatedness to Anxiety				
Total	.033	.059	–.086	.144
Total indirect	.078*	.027	.033	.138
Relatedness → Reappraisal → Anxiety	.061*	.022	.023	.114
Relatedness → Suppression → Anxiety	.016	.016	–.007	.055
Relatedness → Anxiety	–.045	.062	–.170	.074
Autonomy to Assertiveness				
Total	–.019	.061	–.140	.101
Total indirect	.065*	.027	.022	.127
Autonomy → Reappraisal → Assertiveness	.063*	.026	.022	.126
Autonomy → Suppression → Assertiveness	.002	.006	–.004	.024
Autonomy → Assertiveness	–.084	.064	–.211	.043

Supplementary Table 4 continues

Supplementary Table 4 continued

Effect	*β*	SE	Bootstrap Bias–Corrected 95% CI (Lower, Upper)	
Relatedness to Assertiveness				
Total	.214*	.067	.087	.347
Total indirect	.062*	.024	.022	.117
Relatedness → Reappraisal → Assertiveness	.055*	.020	.021	.102
Relatedness → Suppression → Assertiveness	.007	.014	–.017	.040
Relatedness → Assertiveness	.152*	.070	.016	.291
Autonomy to Cognitive				
Total	.074	.058	–.035	.194
Total indirect	.025	.019	–.008	.067
Autonomy → Reappraisal → Cognitive	.029*	.017	.005	.071
Autonomy → Suppression → Cognitive	–.004	.008	–.031	.005
Autonomy → Cognitive	.049	.064	–.072	.178
Relatedness to Cognitive				
Total	.157*	.066	.029	.290
Total indirect	.009	.022	–.034	.052
Relatedness → Reappraisal → Cognitive	.026*	.014	.004	.061
Relatedness → Suppression → Cognitive	–.017	.014	–.052	.005
Relatedness → Cognitive	.148*	.066	.021	.281
Autonomy to Bodily–somatic				
Total	.019	.055	–.090	.125
Total indirect	.053*	.024	.013	.105
Autonomy → Reappraisal → Bodily–somatic	.058*	.022	.022	.109
Autonomy → Suppression → Bodily–somatic	–.004	.008	–.032	.005
Autonomy → Bodily–somatic	–.035	.057	–.145	.076
Relatedness to Bodily–somatic				
Total	.174*	.060	.061	.291
Total indirect	.033	.025	–.014	.085
Relatedness → Reappraisal → Bodily–somatic	.050*	.020	.019	.098
Relatedness → Suppression → Bodily–somatic	–.018	.015	–.058	.002
Relatedness → Bodily–somatic	.141*	.062	.022	.269
Autonomy to Motor–behavioral				
Total	.035	.059	–.081	.152
Total indirect	.055*	.025	.017	.120
Autonomy → Reappraisal → Motor–behavioral	.053*	.023	.018	.112
Autonomy → Suppression → Motor–behavioral	.002	.006	–.004	.027
Autonomy → Motor–behavioral	–.020	.066	–.147	.112
Relatedness to Motor–behavioral				
Total	.172*	.069	.036	.305
Total indirect	.054*	.025	.011	.116
Relatedness → Reappraisal → Motor–behavioral	.046*	.019	.016	.094
Relatedness → Suppression → Motor–behavioral	.007	.014	–.016	.042
Relatedness → Motor–behavioral	.119	.071	–.021	.255

Supplementary Table 4 continues

Supplementary Table 4 continued

Effect	*β*	SE	Bootstrap Bias–Corrected 95% CI (Lower, Upper)	
Autonomy to Operational				
Total	.039	.058	–.073	.153
Total indirect	.055*	.024	.016	.112
Autonomy → Reappraisal → Operational	.055*	.023	.020	.112
Autonomy → Suppression → Operational	.000	.005	–.013	.009
Autonomy → Operational	–.015	.064	–.139	.110
Relatedness to Operational				
Total	.175*	.066	.044	.300
Total indirect	.047*	.024	.004	.101
Relatedness → Reappraisal → Operational	.048*	.019	.017	.092
Relatedness → Suppression → Operational	.000	.013	–.030	.024
Relatedness → Operational	.128	.071	–.012	.263
Autonomy to Social support				
Total	.098	.053	–.005	.203
Total indirect	.041*	.020	.011	.090
Autonomy → Reappraisal → Social support	.039*	.018	.011	.084
Autonomy → Suppression → Social support	.002	.006	–.004	.028
Autonomy → Social support	.057	.055	–.047	.168
Relatedness to Social support				
Total	.435*	.060	.311	.542
Total indirect	.044*	.021	.009	.093
Relatedness → Reappraisal → Social support	.034*	.015	.011	.072
Relatedness → Suppression → Social support	.010	.013	–.010	.044
Relatedness → Social support	.391*	.063	.259	.505
Competence to Communicative				
Total	–.057	.072	–.197	.085
Total indirect	–.115*	.045	–.222	–.043
Autonomy → Reappraisal → Communicative	–.011	.012	–.050	.003
Autonomy → Suppression → Communicative	–.104*	.043	–.208	–.035
Autonomy → Communicative	.058	.071	–.079	.197
Relatedness to Communicative				
Total	–.182*	.064	–.307	–.056
Total indirect	–.100*	.038	–.186	–.034
Relatedness → Reappraisal → Communicative	–.016	.015	–.054	.006
Relatedness → Suppression → Communicative	–.084*	.034	–.162	–.028
Relatedness → Communicative	–.082	.065	–.207	.046

Note. Only results for significant basic needs–modality paths are included. *Significance indicated via 95% CI. Abbreviations: *β* = standardized estimate; SE = Standard error; CI = Confidence interval.
